# Folate metabolism genetic polymorphisms and meningioma and glioma susceptibility in adults

**DOI:** 10.18632/oncotarget.18986

**Published:** 2017-07-04

**Authors:** Dongming Chen, Jun Dong, Ying Huang, Feng Gao, Xiaopeng Yang, Xianglun Gong, Xiaochen Lv, Chenghao Chu, Yonggang Wu, Yong Zheng

**Affiliations:** ^1^ Neurosurgery Department, People’s Hospital of Xinjiang Uyghur Autonomous Region, Urumqi 830001, Xinjiang, China; ^2^ Respiratory Department, People’s Hospital of Xinjiang Uyghur Autonomous Region, Urumqi 830001, Xinjiang, China; ^3^ Nursing College of Xinjiang Medical University, Urumqi 830001, Xinjiang, China; ^4^ Tumor Hospital of Xinjiang Medical University, Urumqi 830001, Xinjiang, China; ^5^ Neurosurgery Department, The Eighth People’s Hospital of Shenzhen, Shenzhen 518101, Guangdong, China

**Keywords:** glioma, meningioma, MTHFR, MTRR, SNP: meta-analysis

## Abstract

Polymorphic variants of genes involved in folate metabolism are implicated in the susceptibility to meningioma and glioma, but the results from published articles are controversial and inconclusive. Therefore, we performed this meta-analysis including all studies available to evaluate the relationship between folate metabolism genetic polymorphisms and the susceptibility to meningioma and glioma in adults. We searched the literature in PubMed, EMBASE and Cochrane Central Library for relevant articles published up to August 2016. The odds ratios (ORs) and the corresponding 95% confidence intervals (95%Cls) were used to evaluate the associations of two folate metabolism genetic variants MTRR A66G (rs1801394) and MTHFR A1298C (rs1801131) with the risk of meningioma and glioma in adults. We found significant association of MTHFR A1298C (rs1801131) variant genotypes with increased incidence of meningioma and glioma in this study population (CA vs. AA: OR=1.22, P<0.001; CA+CC vs. AA: OR=1.18, P=0.002). Moreover, we found that MTRR A66G (rs1801394) variant genotypes was associated with increased risk of meningioma and glioma (G vs. A: OR=1.11, P=0.020; GG vs. AA+AG: OR=1.17, P=0.043; GG vs. AA: OR=1.22, P=0.023). In conclusion, our meta-analysis suggests that two folate metabolism genetic variants MTRR A66G (rs1801394) and MTHFR A1298C (rs1801131) contribute to genetic susceptibility to meningioma and glioma in adults.

## INTRODUCTION

Based on the GLOBOCAN2012 investigations, approximately 14.1 million new cancer cases and 8.2 million deaths were reported worldwide [1]. The overall incidence of brain tumor is estimated at 3.5 case per 100,000 persons, and glioma and meningioma are the most common types of primary brain tumors, accounting for approximately 50% and 20%, respectively [2, 3]. Primary brain tumors mostly occur in familial aggregation, indicating important role of genetic variants in the pathogenesis of brain tumor [4].

Folate metabolism plays an important role in carcinogenesis, due to its involvement in DNA synthesis, methylation and repair. Folate metabolism regulates nucleotide synthesis and DNA methylation via a complex pathway involving at least 30 different enzymes [5]. Therefore, individual genetic variation in these enzymes could change the general balance between DNA synthesis, methylation, and repair. The genes encoding enzymes involved in folate metabolism display several single nucleotide polymorphisms, such as methylenetetrahydrofolate reductase (MTHFR A1298C) and methionine synthase reductase (MTRR A66G). Genetic polymorphisms of folate metabolism pathways have been shown to be associated with diverse tumor types, including pancreatic cancer [6], cervical intraepithelial neoplasia [7], breast cancer [8] and acute leukemia [9–11].

Semmler et al. reported the first case-control study showing that A1298C genetic variant was not significantly associated with brain tumor susceptibility [12]. Up to now, the two most common Folate Metabolism genetic variants A1298C (rs1801131) and A66G (rs1801394) have been studied for their associations with brain tumor susceptibility, but the results from published articles are controversial and inconclusive [12–16]. We hypothesized that the inconsistent results may have been caused by either the relatively small sample sizes of single studies or the genetic heterogeneity of folate metabolism genetic variants in different populations. Therefore, we performed this meta-analysis including all studies available to evaluate the relationship between folate metabolism genetic polymorphisms and the susceptibility to meningioma and glioma in adults. To our knowledge, this is the first comprehensive and systematic meta-analysis to investigate the relationship between genetic polymorphisms of folate metabolism and meningioma and glioma susceptibility in adults.

## RESULTS

### Characteristics of eligible publications

**Figure 1:**
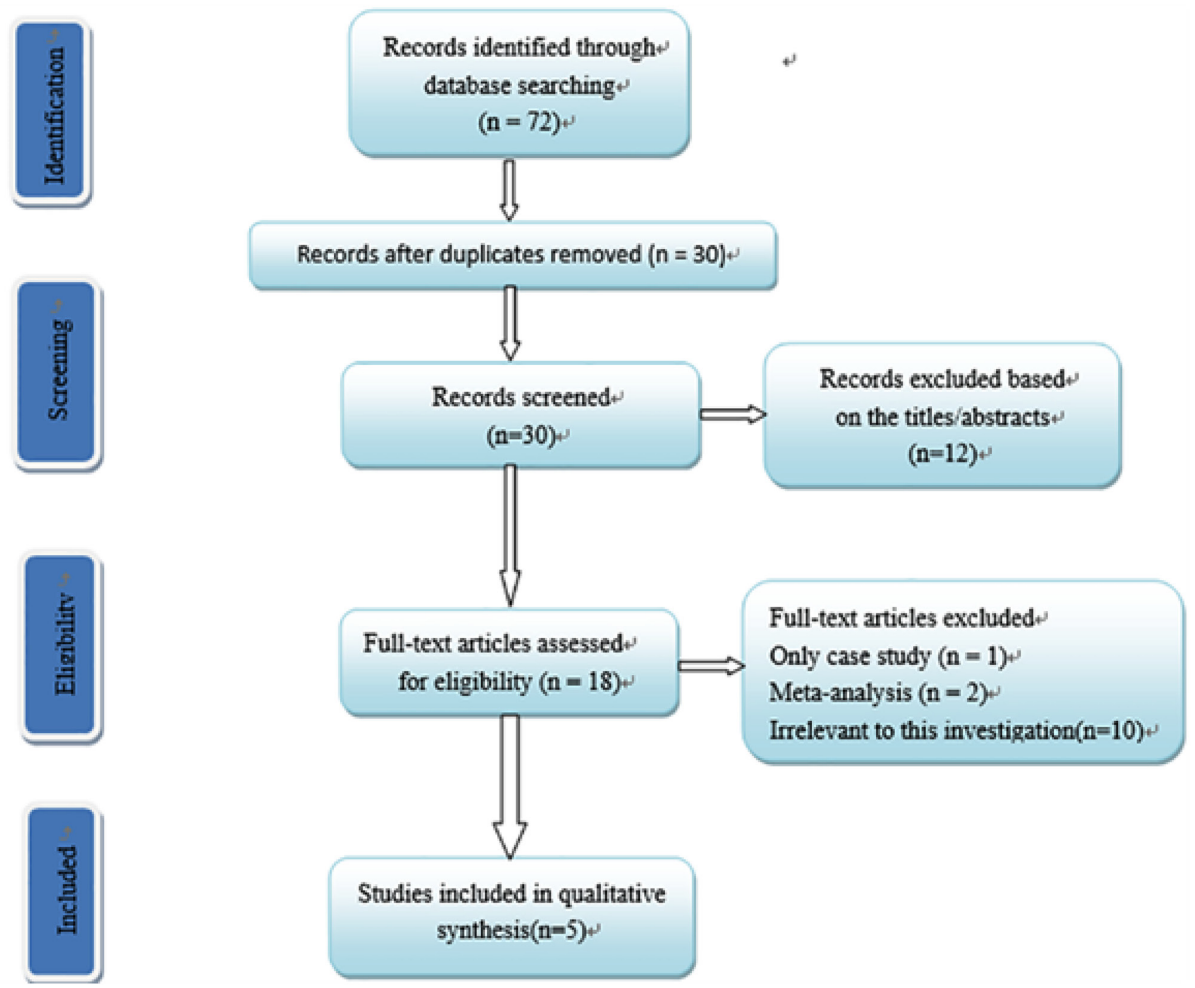
Flow chart of study selection in this meta-analysis

After screening the abstracts, titles, or contents through EMBASE, PubMed and the Cochrane Library, we identified 72 potentially relevant studies and selected five published studies [12–16]. The flow diagram describing the selection of the studies is shown in Figure 1. All selected studies were case-control study, their population size ranged from 154 to 1,200, and were published from 2006 to 2013. All SNPs tested indicated that genotype frequencies in the controls are consistent with the HWE (P > 0.001). The characteristics of the selected studies are summarized in Table 1.

**Table 1:** Main characteristics of studies included in the meta-analysis

	Author	Year	Country	Ethnicity	Cancer type	Control source	Genotyping methods	Case	Control	HWE
A1298C										
	Semmler	2008	German	Caucasian	Meningioma	PB	PCR-RFLP	100	100	0.842
	Zhang	2013	China	Asian	Meningioma	PB	PCR-RFLP	600	600	0.199
	Bethke	2008	UK-North	Caucasian	Meningioma	PB	lllumina	173	175	0.219
	Bethke	2008	UK-Southeast	Caucasian	Meningioma	PB	lllumina	121	123	0.423
	Bethke	2008	Sweden	Caucasian	Meningioma	PB	lllumina	149	149	0.759
	Bethke	2008	Denmark	Caucasian	Meningioma	PB	lllumina	110	113	0.104
	Bethke	2008	Finland	Caucasian	Meningioma	PB	lllumina	77	77	0.783
	Li	2013	China	Asian	Meningioma	PB	PCR-RFLP	317	320	0.063
	Bethke	2008	UK-North	Caucasian	Glioma	PB	lllumina	369	369	0.029
	Bethke	2008	UK-Southeast	Caucasian	Glioma	PB	lllumina	211	214	0.564
	Bethke	2008	Sweden	Caucasian	Glioma	PB	lllumina	197	196	0.495
	Bethke	2008	Denmark	Caucasian	Glioma	PB	lllumina	99	100	0.798
	Bethke	2008	Finland	Caucasian	Glioma	PB	lllumina	128	131	0.746
	Liu	2013	China	Asian	Glioma	HB	PCR	273	326	0.008
A66G										
	Zhang	2013	China	Asian	Meningioma	PB	PCR-RFLP	600	600	0.765
	Bethke	2008	UK-North	Caucasian	Meningioma	PB	lllumina	174	175	0.733
	Bethke	2008	UK-Southeast	Caucasian	Meningioma	PB	lllumina	121	123	0.756
	Bethke	2008	Sweden	Caucasian	Meningioma	PB	lllumina	149	149	0.641
	Bethke	2008	Denmark	Caucasian	Meningioma	PB	lllumina	110	113	0.9
	Bethke	2008	Finland	Caucasian	Meningioma	PB	lllumina	77	77	0.361
	Bethke	2008	UK-North	Caucasian	Glioma	PB	lllumina	128	131	0.212
	Bethke	2008	UK-Southeast	Caucasian	Glioma	PB	lllumina	370	369	0.966
	Bethke	2008	Sweden	Caucasian	Glioma	PB	lllumina	211	214	0.477
	Bethke	2008	Denmark	Caucasian	Glioma	PB	lllumina	197	197	0.872
	Bethke	2008	Finland	Caucasian	Glioma	PB	lllumina	99	100	0.017

PB, population-based; HB, hospital-based; HWE, Hardy-Weinberg equilibrium.

### Association between A66G polymorphism and the susceptibility of meningioma and glioma in adults

**Figure 2:**
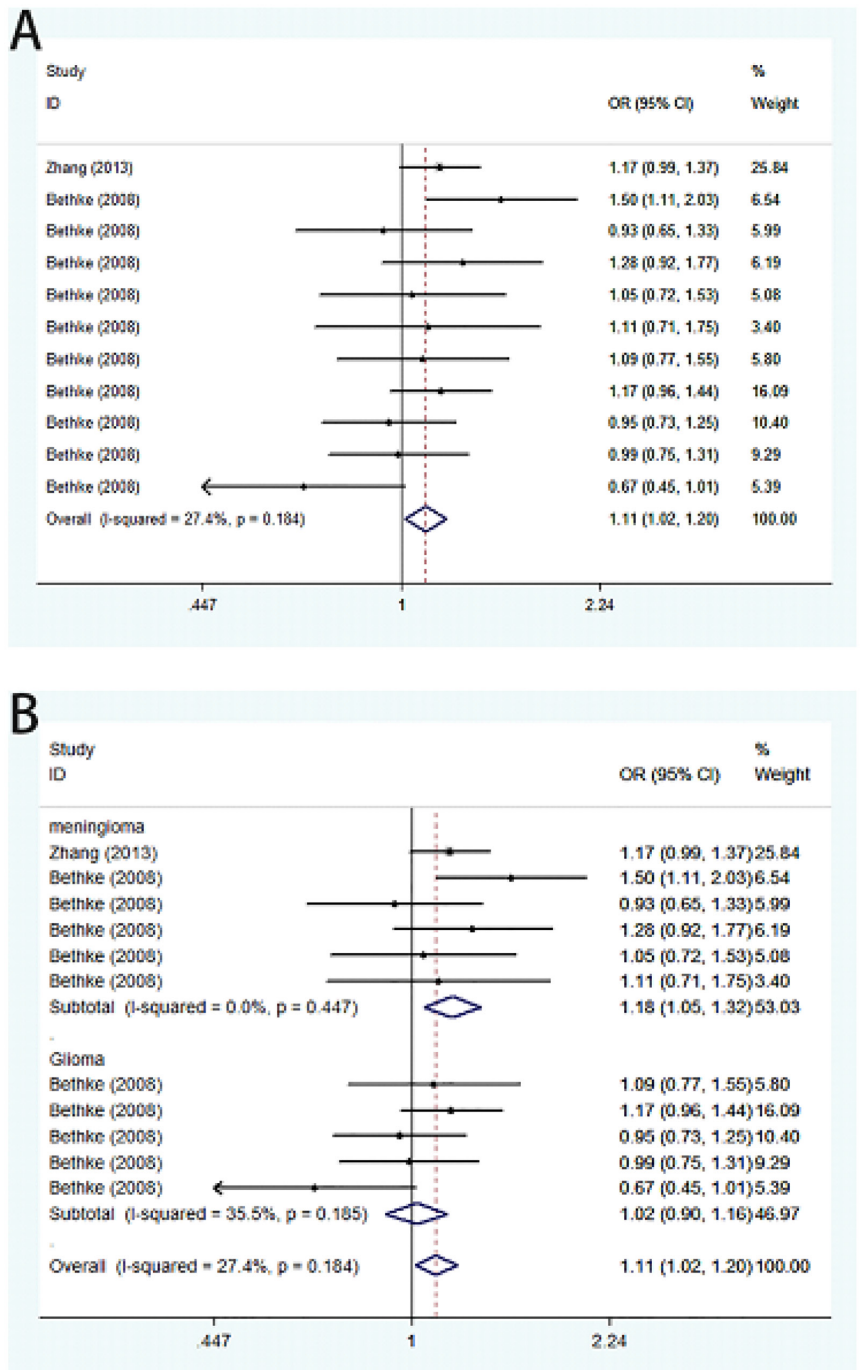
Forest plot on the association between A66G (rs1801394) and meningioma and glioma susceptibility in adults in the allele model **(A)** Overall analysis. **(B)** Subgroup analysis by cancer type.

Meta-analysis of A66G polymorphism in 2,236 cases and 2,248 controls showed a significant association between A66G and the risk of meningioma and glioma (G vs. A: OR=1.11, 95%CI=1.02-1.20; GG vs. AA: OR=1.22, 95%CI=1.03-1.45; GG vs. AA+AG: OR=1.17, 95%CI=1.00-1.36) (Figure 2). Stratification analysis by tumor type showed a significant association of A66G polymorphism with meningioma (G vs. A: OR=1.18, 95%CI=1.05-1.32; GG vs. AA: OR=1.41, 95%CI=1.12-1.77; GG vs. AA+AG: OR=1.32, 95%CI=1.07-1.63; GG+AG vs. AA: OR=1.19, 95%CI=1.01-1.40), but not with glioma. We also implemented stratified analysis by ethnicity, and found a significant association in Asian population (GG vs. AA: OR=1.41, 95%CI=1.02-1.96) (Table 2).

**Table 2:** Meta-analysis of the association between A66G polymorphism and brain tumor susceptibility in adults

Comparison	Subgroup	Studies	Heterogeneity test		Association test		Model	Publication bias	
			P Value	I^2^ (%)	OR (95%CI)	P Value		Begg	Egger
G vs. A	Overall	11	0.184	27.4	**1.11(1.02-1.20)**	**0.02**	F	0.35	0.262
	Meningioma	5	0.447	0	**1.18(1.05-1.32)**	**0.004**	F		
	Glioma	6	0.185	35.5	1.02(0.90-1.16)	0.748	F		
	Caucasian	10	0.155	31.7	1.08(0.98-1.19)	0.109	F		
	Asian	1	NA	NA	1.17(0.99-1.37)	0.062	F		
AG vs. AA	Overall	11	0.806	0	1.08(0.95-1.23)	0.235	F	0.755	0.52
	Meningioma	5	0.447	0	1.12(0.94-1.33)	0.226	F		
	Glioma	6	0.891	0	1.04(0.86-1.27)	0.669	F		
	Caucasian	10	0.752	0	1.10(0.95-1.29)	0.205	F		
	Asian	1	NA	NA	1.03(0.80-1.32)	0.836	F		
GG vs. AA	Overall	11	0.089	39	**1.22(1.03-1.45)**	**0.023**	F	0.213	0.178
	Meningioma	5	0.527	0	**1.41(1.12-1.77)**	**0.004**	R		
	Glioma	6	0.054	57.1	0.95(0.62-1.44)	0.801	R		
	Caucasian	10	0.079	41.7	1.16(0.95-1.41)	0.156	F		
	Asian	1	NA	NA	**1.41(1.02-1.96)**	**0.04**	F		
GG+AG vs. AA	Overall	11	0.648	0	1.12(0.99-1.27)	0.074	F	0.876	0.813
	Meningioma	5	0.394	3.5	**1.19(1.01-1.40)**	**0.04**	F		
	Glioma	6	0.836	0	1.04(0.86-1.25)	0.705	F		
	Caucasian	10	0.554	0	1.12(0.97-1.29)	0.132	F		
	Asian	1	NA	NA	1.12(0.89-1.42)	0.336	F		
GG vs. AA+AG	Overall	11	0.083	39.8	**1.17(1.00-1.36)**	**0.043**	F	0.161	0.08
	Meningioma	5	0.707	4.6	**1.32(1.07-1.63)**	**0.009**	R		
	Glioma	6	0.024	64.5	0.93(0.61-1.40)	0.711	R		
	Caucasian	10	0.09	40.2	1.10(0.92-1.31)	0.294	F		
	Asian	1	NA	NA	**1.39(1.03-1.87)**	**0.029**	F		

OR, odds ratio; CI, confidence interval; F, fixed-effects model; R, random-effects model; NA, not available; PB, population-based; HB, hospital-based

### Association between A1298C polymorphism and the susceptibility of meningioma and glioma in adults

**Figure 3:**
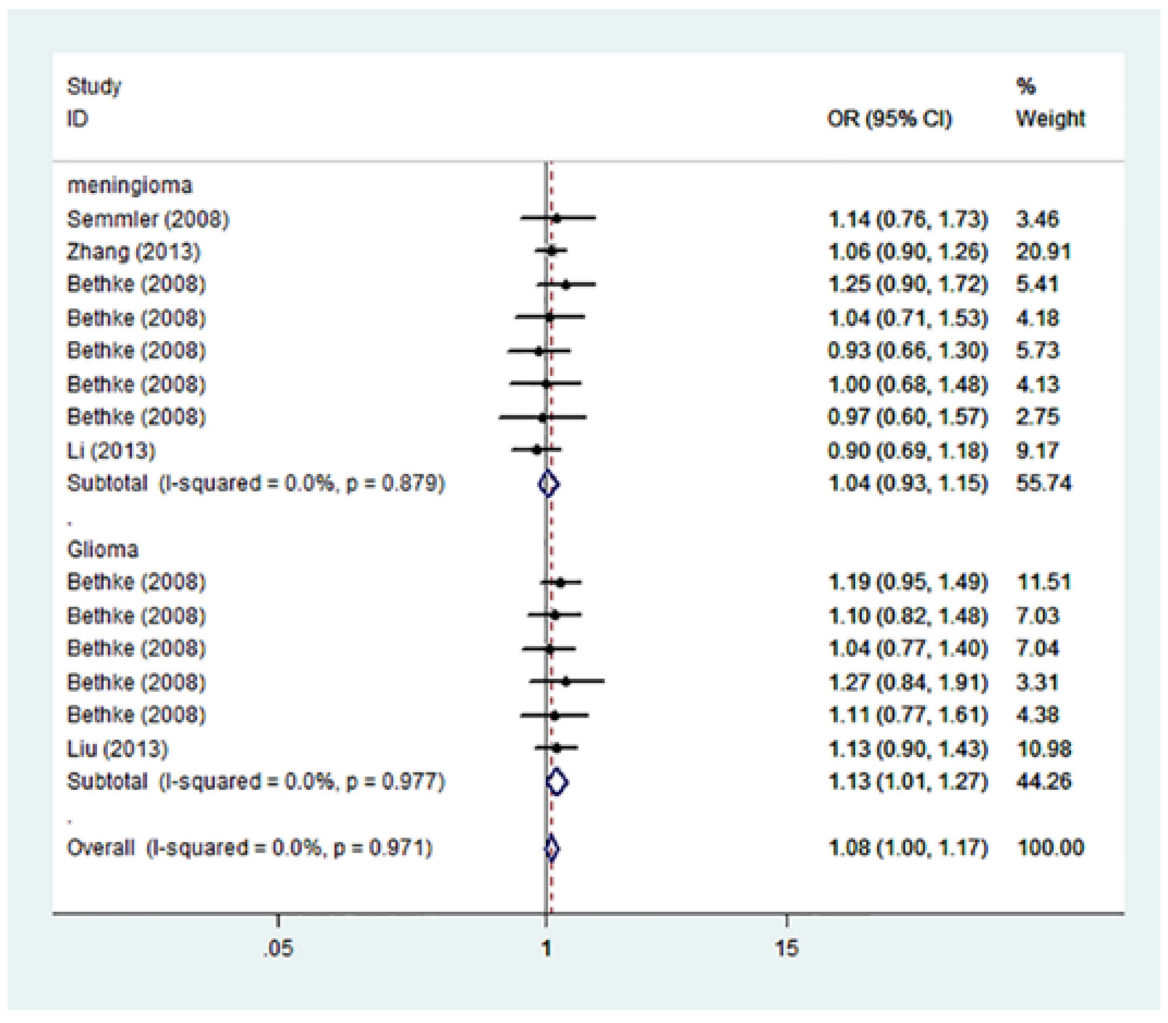
Forest plot on the association between A1298C (rs1801311) and meningioma and glioma susceptibility in adults stratified by cancer type in the allele model

Summary of the association of A1298C polymorphic variant with the risk of meningioma and glioma in adults including 2,997 cases and 3,403 controls is shown in Table 3. Pooled risk evaluation showed a significant association between A1298C and the risk of meningioma and glioma (C vs. A: OR=1.08, 95%CI=1.00-1.17; AC vs. AA: OR=1.22, 95%CI=1.09-1.36; CC+AC vs. AA: 0R=1.18, 95%CI=1.06-1.30) (Figure 3). Then we implemented subgroup analysis by cancer type, and found no significant association of A1298C genotypes with meningioma susceptibility. In contrast, we detected a significantly increased risk of glioma (C vs. A: OR=1.13, 95%CI=1.01-1.27; AC vs. AA: OR=1.35, 95%CI=1.15-1.60; CC+AC vs. AA: OR=1.29, 95%CI=1.11-1.51). Further subgroup analysis showed significantly increased risk of meningioma and glioma in heterozygous model (AC vs. AA: OR=1.19, 95%CI=1.06-1.34), and dominant model (CC+AC vs. AA: OR=1.16, 95%CI=1.04-1.29). We also performed stratified analysis by ethnicity, and found a significant association of A1298C and the risk of meningioma and glioma in heterozygous model (AC vs. AA: OR=1.31, 95%CI=1.14-1.51) and dominant model (CC+AC vs. AA: OR=1.25, 95%CI=1.09-1.42) in Caucasian. However, there was no significant association with the risk of meningioma and glioma in Asian under any genetic model (Table 3).

**Table 3:** Meta-analysis of the association between A1298C polymorphism and brain tumor susceptibility in adults

Comparison	Subgroup	Studies	Heterogeneity test		Association test		Model	Publication bias	
			P Value	I^2^ (%)	OR (95%CI)	P Value		Begg	Egger
C vs. A	Overall	14	0.971	0	1.08(1.00-1.17)	0.056	F	0.743	0.854
	Meningioma	8	0.879	0	1.04(0.93-1.15)	0.522	F		
	Glioma	6	0.977	0	**1.13(1.01-1.27)**	**0.033**	F		
	Caucasian	11	0.978	0	1.10(1.00-1.22)	0.059	F		
	Asian	3	0.447	0	1.05(0.93-1.18)	0.474	F		
	PB	13	0.958	0	1.07(0.99-1.17)	0.097	F		
	HB	1	NA	NA	1.13(0.90-1.43)	0.296	F		
AC vs. AA	Overall	14	0.717	0	**1.22(1.09-1.36)**	**<0.001**	F	0.584	0.189
	Meningioma	8	0.649	0	1.12(0.97-1.30)	0.119	F		
	Glioma	6	0.868	0	**1.35(1.15-1.60)**	**<0.001**	F		
	Caucasian	11	0.981	0	**1.31(1.14-1.51)**	**<0.001**	R		
	Asian	3	0.13	51	1.11(0.86-1.44)	0.413	R		
	PB	13	0.764	0	**1.19(1.06-1.34)**	**0.002**	F		
	HB	1	NA	NA	**1.50(1.05-2.14)**	**0.025**	F		
CC vs. AA	Overall	14	0.941	0	1.03(0.86-1.22)	0.771	F	0.274	0.131
	Meningioma	8	0.636	0	0.98(0.77-1.24)	0.854	F		
	Glioma	6	0.987	0	1.09(0.84-1.40)	0.531	F		
	Caucasian	11	0.916	0	0.99(0.79-1.25)	0.942	F		
	Asian	3	0.51	0	1.07(0.82-1.40)	0.599	F		
	PB	13	0.914	0	1.01(0.84-1.22)	0.883	F		
	HB	1	NA	NA	1.11(0.69-1.77)	0.676	F		
CC+AC vs. AA	Overall	14	0.88	0	**1.18(1.06-1.30)**	**0.002**	F	0.743	0.328
	Meningioma	8	0.836	0	1.09(0.95-1.25)	0.208	F		
	Glioma	6	0.921	0	**1.29(1.11-1.51)**	**0.001**	F		
	Caucasian	11	0.991	0	**1.25(1.09-1.42)**	**0.001**	F		
	Asian	3	0.212	35.5	1.08(0.92-1.27)	0.35	F		
	PB	13	0.893	0	**1.16(1.04-1.29)**	**0.009**	F		
	HB	1	NA	NA	1.38(0.99-1.93)	0.058	F		
CC vs. AA+AC	Overall	14	0.841	0	0.93(0.78-1.09)	0.363	F	0.511	0.085
	Meningioma	8	0.394	4.6	0.92(0.73-1.16)	0.483	F		
	Glioma	6	0.983	0	0.93(0.73-1.19)	0.561	F		
	Caucasian	11	0.844	0	0.87(0.70-1.09)	0.222	F		
	Asian	3	0.416	0	1.00(0.78-1.29)	0.990	F		
	PB	13	0.786	0	0.93(0.78-1.12)	0.448	F		
	HB	1	NA	NA	0.89(0.58-1.36)	0.587	F		

OR, odds ratio; CI, confidence interval; F, fixed-effects model; R, random-effects model; NA, not available; PB, population-based; HB, hospital-based

### Heterogeneity and sensitivity analysis

**Figure 4:**
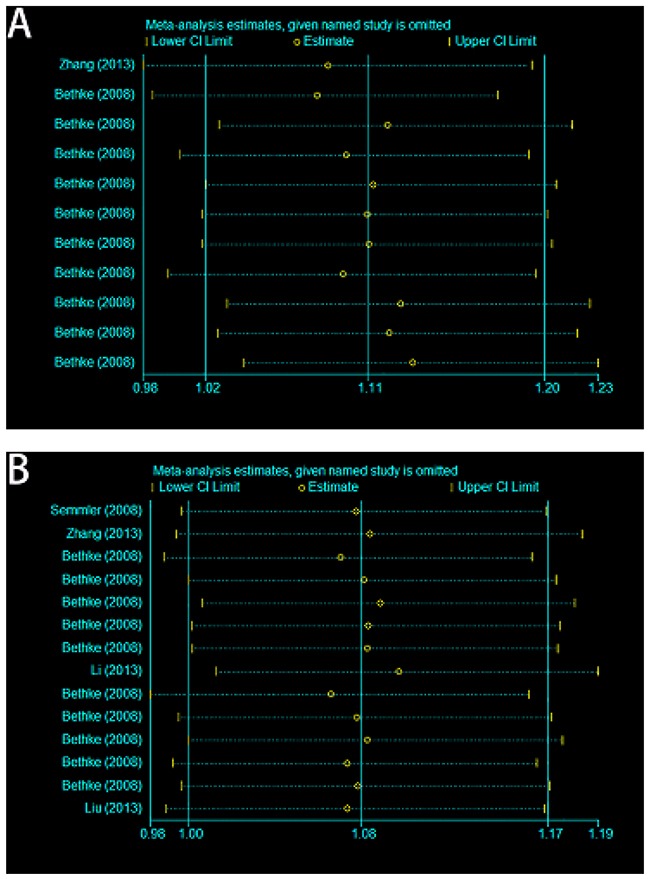
Sensitivity analyses of current meta-analysis in the allele model **(A)** A66G (rs1801394) and meningioma and glioma risk in adults. **(B)** A1298C (rs1801311) and meningioma and glioma risk in adults.

Between-study heterogeneity was calculated by using Q statistics. Fixed-effect model was utilized if p-value of heterogeneity tests was more than 0.05 (P>0.05) [17]; otherwise, the random-effect model was applied [18]. The sensitivity analysis was conducted by omitting each eligible study each time. The pooled ORs for the effects of A66G and A1298C on the risk of meningioma and glioma indicated that our results were statistically robust and stable (Figure 4).

### Publication bias

**Figure 5:**
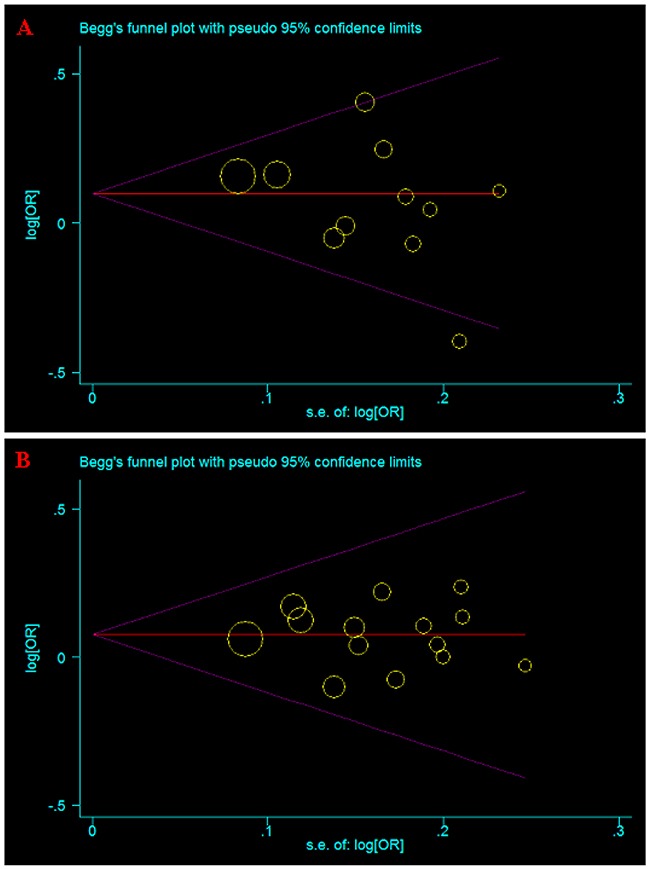
Begg’s funnel plot for publication bias test of current meta-analysis in the allele model **(A)** A66G (rs1801394). **(B)** A1298C (rs1801311).

Both Begg’s and Egger’s tests were used to evaluate the publication bias of the studies [19, 20]. The results showed that there was no obvious publication bias in total population (Table 2 and Table 3, Figure 5A, 5B).

## DISCUSSION

Accumulating evidences indicate that the intake of folic acid has a negative correlation with cancer [21–23], including brain tumors [16]. It is known that folate metabolism will produce intermediate products that participate in nucleotide synthesis, DNA methylation and histone methylation [24–26]. Methylenetetrahydrofolate reductase (MTHFR) and methionine synthase reductase (MTRR) are key enzymes of folate metabolism that are involved in two important branches of folate metabolism: nucleotide synthesis and DNA methylation [24]. The polymorphisms of the genes encoding these enzymes will affect the process of folate metabolism and thus disturb DNA synthesis, repair and methylation, contributing to the occurrence of brain tumor [25].

Human MTHFR gene is located on 1p36.3 locus and contains 11 exons and 10 introns, encoding 656 amino acids [27, 28]. MTHFR protein turns 5,10-methylenetetrahydrofolate into 5-methylenetetrahydrofolate [29]. 5- methylenetetrahydrofolate is a collaborative substrate of homocysteine which is transformed to methionine, and provides methyl groups. MTHFR gene mutation could reduce methylene tetrahydrofolate reductase activity, leading to a higher plasma homocysteine concentration and abnormal metabolism of folic acid, and disturbed DNA synthesis and DNA damage repair [26, 30–33]. MTRR gene is located on the short arm of chromosome 5 (5p15.2-15.3) and contains 15 exons and 14 introns. The most common MTRR mutation is A66G polymorphism, causing the change of isoleucine to methionine, which can reduce enzyme activity and affect the metabolism of homocysteine [32–34].

Although increased number of case-control studies investigated the association of folate metabolism genetic variants with brain tumor susceptibility in adults, the results are inconclusive. Numerous studies have reported that folate metabolism genetic variants are associated with the risk of several cancers, including head and neck [35], lung [36], breast [8], and colorectal cancer [37, 38]. Semmler et al. reported the first case-control study showing that folate metabolism genetic variants was not significantly associated with brain tumor susceptibility [12]. However, Li et al. found that folate metabolism genetic variants may play a pivotal role in the pathogenesis of meningioma [15]. In addition, Bethke et al. reported that genetic variants in folate metabolism affected the risk of developing both meningioma and glioma [16]. To our knowledge, our study is the first comprehensive and systematical meta-analysis to evaluate potential association between two folate metabolism genetic variants (A1298C and A66G) and meningioma and glioma susceptibility in adults [12–16]. Our results showed that A1298C variant significantly increased meningioma and glioma susceptibility in Caucasian. Meanwhile, we observed that A66G variant was associated with increased meningioma and glioma susceptibility in Asian. Our results are consistent with a previous meta-analysis showing that MTRR rs1801394 polymorphism may increase the risk of meningioma [39].

The findings of current study should be interpreted with caution due to several potential limitations. First, the majority of the subjects included in present study were ethnically Caucasian, thus subjects from more diverse ethnicities should be included in future studies. Second, the number of subjects enrolled in certain subgroups was relatively small. Owing to the lack of the original data, we could not estimate meningioma and glioma susceptibility stratified by the gender, age, life-style and other risk factors. Data from large-scale multi-center studies are needed to verify the association between folate metabolism genetic variants (A1298C and A66G) and meningioma and glioma susceptibility in adults.

In conclusion, our findings suggest that folate metabolism genetic variants (A1298C and A66G) may increase the susceptibility of meningioma and glioma in adults. Further large-scale, multi-center and well-designed studies are necessary to investigate the potential function of genetic variation of folate metabolism in meningioma and glioma in adults.

## MATERIALS AND METHODS

### Search strategy

We carried out a literature search in EMBASE, PubMed, and the Cochrane Library up to August 2016. We performed electronic searches using the terms “brain tumor” or “glioma” or “meningioma”, “polymorphism*” or “variant*” or “mutation”, “MTHFR” or “MTRR”.

### Selection criteria

Two authors (GXL. and CCH.) independently screened titles and abstracts to identify relevant studies. Published case-control studies will be included in current meta-analysis if they reach the following criteria: (a) evaluating the association between folate metabolism genetic variants (A1298C and A66G) and brain tumor susceptibility in adults; (b) case-control studies on human, and published in English; (c) sufficient data for assessing the ORs and 95%CI, and P value. Exclusion criteria were: (a) case reports, letters, and review articles; (b) containing only case groups; (c) duplication of published articles.

### Data extraction

Two authors (GXL. and LXC) independently extracted data from all eligible studies. Data such as: (a) the first author name, publication date, country, ethnicity and source of control; (b) cancer types, frequency of cases and controls, involved genes and HWE status in controls. Any disagreements between the two authors were resolved through discussions and agreements

### Statistical analyses

The strength of association between folate metabolism genetic variants (A1298C and A66G) and meningioma and glioma susceptibility in adults was calculated by odds ratios (ORs) with 95% confidence interval (CI). We used dominant, recessive, homozygote, heterozygote, allelic as the models. Stratified analyses were conducted by cancer type, ethnicity and sources of control. The pooled ORs were calculated for these five models. Heterogeneity was evaluated by using Q statistics (significant at p < 0.05). A Fixed-effect model was utilized if p-value of heterogeneity tests were more than 0.05 (P>0.05) [17]; otherwise, the random effect model was applied [18]. The sensitivity analysis was conducted by discarding each eligible study each time to estimate the stability of the results. Potential publication bias was evaluated by Egger’s and Begg’s tests (P < 0.05 was considered significant) [19, 20]. All analyses were conducted by STATA 12.0 (Stata Corp LP, College Station, TX, USA).
